# Electrochemical determination of thiethylperazine using semi-graphitized carbon nanofibers-MnO nanocomposite

**DOI:** 10.1007/s00604-023-06025-1

**Published:** 2023-10-24

**Authors:** Joanna Smajdor, Marcel Zambrzycki, Mateusz Marzec, Beata Paczosa-Bator, Robert Piech

**Affiliations:** 1 Department of Analytical Chemistry and Biochemistry, Faculty of Materials Science and Ceramics, AGH University of Science and Technology, Al. Mickiewicza, 30-059 Krakow, Poland; 2 Department of Biomaterials and Composites, Faculty of Materials Science and Ceramics, AGH University of Science and Technology, Al. Mickiewicza, 30-059 Krakow, Poland; 3 Surface and Biomaterials Nanoengineering, Academic Centre for Materials and Nanotechnology, AGH University of Science and Technology, Al. Mickiewicza, 30-059 Krakow, Poland

**Keywords:** Thiethylperazine, Carbon nanofibers, Carbon nanocomposite, Modified glassy carbon electrode, Differential pulse voltammetry, Amperometry, Flow injection analysis

## Abstract

A new voltammetric method is proposed for high sensitive thiethylperazine (THP) determination, using a glassy carbon electrode modified with semi-graphitized carbon nanofibers/MnO nanocomposite (eCNF/MnO/GC). To the best of our knowledge, this is the first electrochemical assay of THP  determination, and the first use of the eCNF/MnO as the electrode modifier. The proposed method using eCNF/MnO/GC is characterized by high repeatability and sensitivity of measurements, with the linearity of THP in the range from 0.05 to 2.2 µmol L^−1^. The lowest detection limit achieved on the eCNF/MnO/GC electrode for 30 s of preconcentration was 6.3 nmol L^−1^ THP in 0.05 mol L^−1^ acetate buffer of  pH 5.6. The proposed method was successfully applied to highly sensitive THP determination in complex matrices, such as tablets and plasma with good recovery (98–103%). The RSD value obtained for THP measurement at a concentration of 0.1 µmol L^−1^ was 1.3%. Amperometric measurements of THP under the flow injection conditions were also performed to indicate the possibility of its fast and accurate determination (103% and 95% for unmodified and modified electrode, respectively), with the duration of single analysis of approx. 30 s.

**Graphical Abstract:**

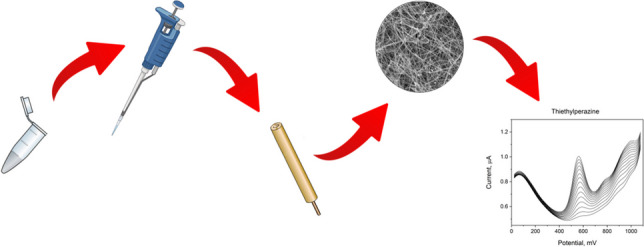

**Supplementary Information:**

The online version contains supplementary material available at 10.1007/s00604-023-06025-1.

## Introduction

Thiethylperazine (THP) is a phenothiazine derivative, an antagonist of dopaminergic receptors. It has an antiemetic effect by affecting the vomiting center in the reticular formation of the medulla oblongata and the chemoreceptor zone that triggers the gag reflex in the fourth ventricle of the brain [1]. It is likely that the drug also inhibits the afferent impulses of the autonomic nervous system via the vagus nerve [2]. Thiethylperazine under the brand name Torecan® is used in the treatment of conditions such as nausea and vomiting after surgery, anti-cancer chemotherapy, radiotherapy, or treatment with toxic drugs [3–5]. It is well absorbed after oral administration. It is very lipophilic and highly bound to plasma membranes and proteins. It accumulates in organs with high blood flow and easily crosses the placenta. Metabolism occurs mainly in the liver, only 3% is excreted unchanged by the kidneys, excreted mainly in the urine, mainly in the form of metabolites [6]. The pharmacokinetics of thiethylperazine in humans have not been thoroughly studied.

There are not many literature reports on the quantification of thiethylperazine. Among the available papers, the fluorimetric [7] and spectrophotometric [8] methods have been published. Furthermore, mass spectrometry [9, 10] and high-performance liquid chromatography and gas chromatography coupled with surface ionization and mass spectrometry detectors [11, 12] have been used for highly sensitive determination of thiethylperazine. However, no electrochemical methods have ever been reported for this purpose. Electroanalytical methods of analysis allow us to avoid such inconvenience of measurements such as a long time of analysis or requirements of expensive aparature, while ensuring low detection limits, high sensitivity, wide linear range, and simple procedure of sample analysis. Different voltammetric techniques and types of working electrodes are used in this purpose. One of the most popular electrodes for the determination of organic compounds is the glassy carbon electrode. It is characterized by easiness of preparation, and can be modified by a wide variety of mixtures. One of the most popular modifiers are different carbon materials, e.g., carbon nanotubes, carbon black, graphene, carbon nanofibers etc. [13–15].

Electrospun carbon nanofibers (eCNF) and their hybrids with metal and oxide nanoparticles are especially useful for this purpose due to the number of advantages predisposing them to applications related to electrochemistry. In such nanocomposite systems, eCNFs are usually used as a support providing electrically conductive network of fibers with large surface area, in which the nanoparticles are anchored, ensuring number of well available catalytic active sites. Carbon nanofibers possess a high share of sp^2^ bonds providing good electron transport, and their elongated shape is especially beneficial considering percolation and electrical contact between nanofibers and nanoparticles. Importantly, eCNFs are much cheaper than carbon nanotubes or graphene. In turn, manganese oxide nanoparticles are one of the inorganic nanostructures willingly used in hybrids with the carbon nanomaterials, with the aim of improving their electrochemical activity and providing catalytic sites. Manganese oxides are characterized by high electrocatalytical activity, a quite high energy density, low cost, an environmental friendliness, nontoxicity, abundance, etc. [16, 17]. Furthermore, manganese oxides can simultaneously act as a graphitization catalyst leading to increased electrical conductivity of carbon nanofibers, which adds to the potential list of benefits of their use together with eCNF [18, 19]. Until now, the majority of the work in this field were majorly devoted primarily to other types of manganese oxides: α-MnO_2_, β-MnO_2_, α-Mn_2_O_3_, Mn_3_O_4_, relatively rarely investigating MnO and its nanocomposites [17, 20, 21]. One of the recently demonstrated applications of MnO was its hybrid with N-doped carbon nanofibers proposed by Yen et al. as an interlayer material for lithium batteries [18]. In other work, Ding et al. developed an advanced MnO/carbon network catalyst for direct hydrazine fuel cells, which showed significantly enhanced electrocatalytical activity [22]. No work on eCNF/MnO nanocomposites for electrochemical detection of drugs has been found; however, some types of CNFs were used before in this case [23–25].

In this work, a simple voltammetric assay is proposed for high sensitive thiethylperazine determination. As a working electrode, a glassy carbon electrode modified with semi-graphitized carbon nanofibers/MnO nanocomposite (eCNF/MnO/GC) was used. To the best of our knowledge, this has been the first time that THP is measured using an electrochemical method, and such modifier is used for the voltammetric assay.

## Materials and methods

### Apparatus

The electrochemical analyzer type M161 with the electrode stand type M164 purchased from mtm-anko (Warsaw, Poland) was used for voltammetric measurements. In the measurement cell with the volume of 20 mL, an Ag/AgCl/KCl (3 mol L^−1^) electrode with the replaceable outer cover (3 mol L^−1^) was used as a reference electrode, the platinum wire was used as the auxiliary electrode, and the glassy carbon electrode modified with eCNF/MnO nanocomposite was used as a working electrode. Voltammograms were registered and then visualized using dedicated EAQt software, further analysis and visualization of data was performed with Origin Lab 2021b. During the preconcentration of the analyte, stirring was performed by the Teflon coated magnetic bar with the approx. speed of 500 rpm. pH measurements were performed using the Elmetron CX-705 (Zabrze, Poland).

The flow system consisted of a 0.05-L solution reservoir, 800 Dosino pump with 900 Touch control panel (both Metrohm, Switzerland) and a Rheodyne Model 7010 sample injection valve with sample loop of 100 μL and the flow detector. All interconnections were made of 0.5 mm Tygon tubing. The length of tubing between the valve and the detector was 1 m. The wall–jet detector was used in the form of the screen printed carbon electrodes with built in reference electrode (Metrohm, Switzerland).

The electrical impedance spectroscopy measurements (EIS) were conducted with the use of an VersaSTAT 4 Potentiostat Galvanostat (Princeton Applied Research, Ametek Scientific Instruments, USA). Studied GC electrodes modified with different modifiers were connected for measurements as working electrodes into a three-electrode cell with an Ag/AgCl/KCl (3 mol L^−1^) electrode as the reference electrode and a glassy carbon rod as the auxiliary one. All data analysis was carried out and interpreted using ZSimpWin 3.60 software. Measurements were performed in 1 mmol L^−1^ K_3_[Fe(CN)_6_] in 1 mol L^−1^ KCl using the sinusoidal signals of frequency ranging from 100 kHz to 10 mHz and 50 mV amplitude, superimposed on the formal potential of K_3_[Fe(CN)_6_].

### Chemicals and glassware

All materials were purchased with the producers purity and used without any additional purification. Standard stock solution of 0.01 mol L^−1^ thiethylperazine maleate (99.85%, LGC, Germany) was prepared by dissolving it in double distilled water and ethanol (5:1) and stored in the fridge. Solutions with lower THP concentration were prepared daily. Acetate buffer (0.5 mol L^−1^, pH 5.6) was prepared by mixing the corresponding amounts of glacial acetic acid (Suprapur, Aldrich) and crystalline sodium acetate anhydrous (> 99%, Aldrich). Hydrochloric acid (Suprapur, 30%) was purchased from Merck (Germany). Double distilled water was used to prepare all solutions.

For the synthesis of nanocomposite, polyacrylonitrile M_w_ = 150 000 Da (PAN, Millipore Sigma), manganese (III) acetylacetonate (Mn(Acac)_3_, 97% Acros Organics), dimethylformamide (DMF, ≥ 99.8%, Avantor Poland), and nitrogen (Air Liquide Poland) were used. All of chemicals were of analytical grade.

### Sample preparation

#### Tablet

Thiethylperazine concentration was measured in pharmaceutical formulation Torecan (Krka, 6.5 mg thiethylperazine in the form of thiethylperazine maleate per tablet) (Slovenia) purchased in the local drugstore. For the quantitative analysis, three of them were crushed in the mortar, dissolved in the volumetric flask with double distilled water and ethanol (5:1), and then placed in the ultrasonic bath for 5 min (Intersonic IS-1 K, Poland). Obtained suspension before measurements was filtered using syringe filters with the membrane made from regenerated cellulose and pore size of 0.45 μm (Biosens, Poland).

#### Plasma

Plasma sample was obtained from Biowest (France) and stored in the freezer at -20 °C, according to the manufacturer recommendation. Before the measurements, 800 μL of plasma was mixed with 200 μL of 10% trichloroacetic acid (TCA), next shaken for 2 min on the vortex (Biosan, Poland) and then centrifuged for 30 min with an approximate speed of 10 000 rpm (Eppendorf, Germany). Obtained supernatant was filtered on the syringe filters with the RC membrane and pore size of 0.45 μm (Biosens, Poland) and used in further measurements.

### Preparation of eCNF/MnO

The precursor solution for the electrospinning was prepared by adding 1 g of PAN dried overnight to 10 g of DMF and the resulting mixture was placed on a magnetic stirrer for 24 h. Afterwards, the solution was transferred into plastic syringe with steel needle (d = 1.1 mm) used as a spinneret in custom made electrospinning set-up with rotary collector. The conditions of the electrospinning were as follows: U = 5.5 kV; distance to collector = 40 mm; t = 40 min; T ~ 25 °C; gravitational outflow. As a result, thin sheets of PAN nanofiber non-wovens were obtained, which in the next step were annealed in the ambient atmosphere from RT to 270 °C and held in this temperature for 30 min in order to pre-oxidize PAN [26]. Subsequently, 4 mg of pre-oxidized nanofibers was put on Teflon plate, and subjected to impregnation with 150 μL 5% solution of Mn(Acac)_3_ in DMF. Dried material was then transferred to the tubular furnace and heated up to 1000 °C and annealed for 10 min with aim to carbonize the nanofibers and convert Mn(Acac)_3_ to manganese oxides. During the treatment, the reactor was purged with N_2_. Finally, the obtained electrospun carbon nanofibers/MnO (eCNF/MnO) nanocomposite was dispersed in DMF in concentration of 1 mg ml^−1^ using ultrasonic processor, and the resulting suspension was used for the further depositions on GC electrodes.

### Working electrode preparation

Voltammetric measurements were performed on the glassy carbon electrode with the diameter of 3 mm (Mineral, Poland). The surface of the electrode was prepared for modification by polishing on the polishing pad and using alumina slurry with the particle size of 0.3 μm (Buehler, USA). After rinsing the leftovers from the Al_2_O_3_ powder, electrode was placed in the beaker with the methanol and placed in the ultrasonic bath (Intersonic IS-1 K, Poland) for 3 min. Working electrode was modified with the 2.5 μL eCNF/MnO suspension, left to dry for approx. 3 h, and then used in the measurements. Mass loading of the modifier on single electrode was 2.5 μg.

For the measurements performed using flow injection analysis, screen-printed carbon electrode (Methrom, Switzerland) was used without any further preparation.

### Characterization of eCNF/MnO nanocomposite

#### Scanning electron microscopy and energy-dispersive X-ray spectroscopy

The microstructure of the samples was examined using scanning electron microscope (SEM) ThermoFischer Scientific Phenom XL Desktop. Observations were carried out in high vacuum conditions using secondary electron and back-scattering electron detectors. The accelerating voltage was set to 10 kV. During observations, the chemical composition of the samples was analyzed with energy dispersive X-ray spectroscope (EDS, Phenom) coupled with the microscope.

#### X-ray diffraction

The phase composition and structure of samples were investigated through X-ray diffraction (XRD) using Panalytical X′Pert Pro X-ray diffractometer working with Cu Kα source (λ = 1.5406 nm). The measurements were performed in the grazing incidence diffraction (GID) mode, in the 2θ range of 15–60° and with angular resolution of 0.02°. The values of interplanar distance d_hkl_, and mean size of coherent domains L_c_ were calculated using Bragg’s and Sherrer’s equations [27].

#### X-ray photoelectron spectroscopy

The surface chemistry and valence state of present elements were analyzed using X-ray photoelectron spectroscopy (XPS) at PHI VersaProbe II Scanning XPS with monochromatic Al Kα X-ray source (1486.6 eV). The measurements were carried out in ultra-high vacuum conditions, with electron take-off angle of 45°, and pass energy set to 117.50 eV for survey scan, and 46.95 eV for core level spectra. Collected data was calibrated with reference to C1s line set to 285.0 eV, and subjected to background subtraction using Shirley method.

#### Raman spectroscopy

The complementary information on the structure of nanocomposites was obtained using Raman spectrometer Horiba LabRAM HR equipped with 532 nm laser source, and Olympus Plan M NA09 lens. Spectra were collected within range of 50 to 4000 cm^−1^, with integration time of 10 s, and resolution of 0.39 cm^−1^. Four spectra were collected for each sample, and calculated parameters were averaged.

### Measurement procedure

Thiethylperazine quantitative analysis was performed using the differential pulse voltammetry (DPV) technique. Measurements were conducted in the 0.05 mol L^−1^ acetate buffer with pH 5.6 as a supporting electrolyte. The instrumental parameters of the high sensitive THP measurements were as follows: sampling and waiting time t_p_ = t_w_ = 10 ms, step potential E_s_ = 5 mV and pulse amplitude dE = 50 mV. The voltammograms were registered in the potential range from 0 to 1100 mV, after the preconcentration step described by the parameters: E_acc_ = 0 mV and t_acc_ = 5 s. Between each voltammogram registration, a rest period of 20 s was strictly required in order to obtain the high repeatability of the THP signals.

## Results

### Characterization of eCNF/MnO nanocomposite

#### Morphology

**Fig. 1:**
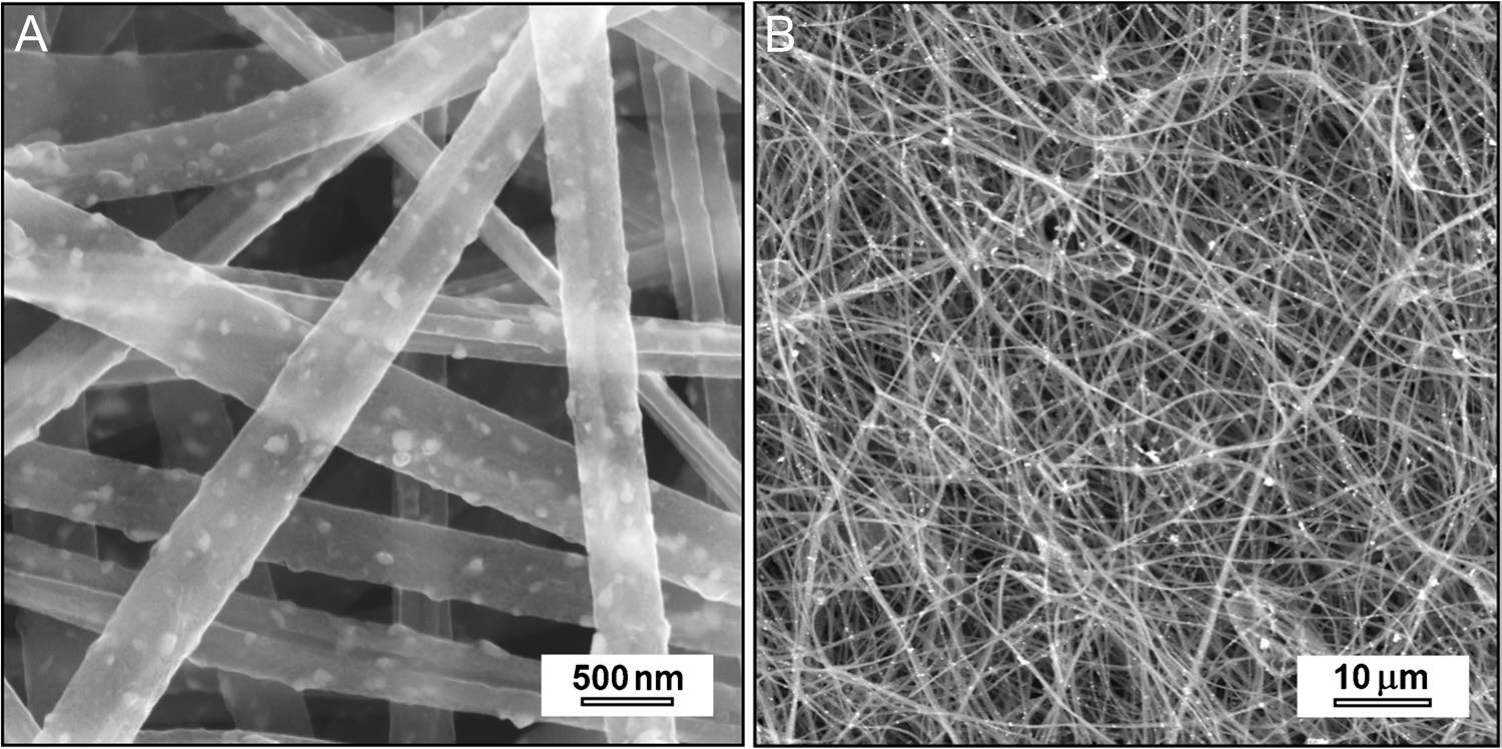
SEM images of eCNF/MnO nanocomposite. Magnifications: **A** 25 000x. **B** 2000x

The SEM images of eCNF/MnO nanocomposite are shown in Fig. 1. Performed observations revealed correct, fibrous morphology of samples, and showed no visible defects of nanofibers nor particle aggregates. The fibers had mean diameter of 311 nm, and were covered with evenly distributed fine nanoparticles formed as a product of thermal decomposition of Mn(Acac)_3_. The sizes of nanoparticles ranged mainly between 30 and 90 nm, thus ensuring well accessible, large surface area for electrochemical reactions to take place.

#### Phase composition and structure

**Fig. 2:**
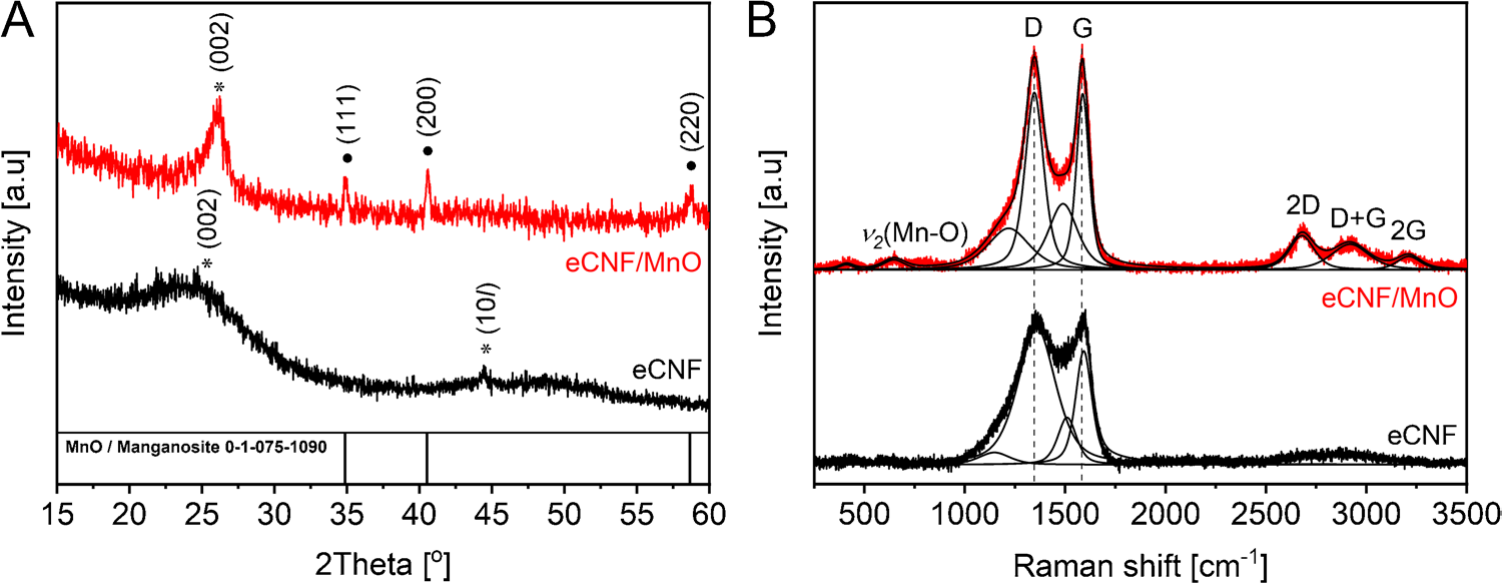
XRD patterns (**A**) and Raman spectra (**B**) of eCNF/MnO nanocomposite compared to reference eCNF. (*—turbostratic carbon, eCNF; •—fcc-MnO (*manganosite*)). *—Raman spectra of pure MnO can be found in the Supplementary Information [48]

The X-ray diffractograms of eCNF/MnO nanocomposite and reference, non-treated eCNF are shown in Fig. 2A. The XRD pattern of nanocomposite demonstrated peak at 26.1° originating from (002) planes of turbostratic carbon, and three reflexes at 34.9, 40.6, and 58.7° attributed to (111), (200) and (220) planes of fcc-manganese (II) oxide (*Fm-3 m*) (ICSD 030520—*manganosite*) [28]. No lines characteristic for other types of manganese oxides were detected. In addition, the comparison of XRD patterns of eCNF/MnO and non-treated eCNF revealed significant shift, and narrowing of the (002) peak, which indicate a catalytical graphitization of carbon induced by MnO, as evidenced by the decreasing interplanar distance d_002_ from 0.363 to 0.341 nm, and increasing size of carbon crystallites L_c_ from 1.1 to 5.1 nm [29]. This phenomenon is related to growth of graphitic crystalline domains and ordering of carbon structure, which results in enhancement of its electrical conductivity, thus being potentially beneficial for the electron transport in a electrochemical sensor devices [30, 31]. As the temperature of heat treatment was too low to fully transform the structure of into graphitic carbon, obtained materials can be considered as semi-graphitized. The value of degree of graphitization calculated from DOG = (0.3440-d_002_)/(0.3440–0.3354) × 100% for eCNF/MnO was 34.9%, thus confirming semi-graphitic structure of carbon [32].

In the next step, Raman spectroscopy measurements were carried out to provide further information regarding the structure of samples and the results are shown in Fig. 2B. The spectrum of eCNF/MnO nanocomposite was characterized by the presence of very weak ν_2_(Mn–O) feature at 645 cm^−1^ assigned to Mn–O A_1g_ breathing modes, and strong peaks originating from sp^2^ carbon: D (1350 cm^−1^) and G (1590 cm^−1^) bands, and second order lines – 2D (2680 cm^−1^), D + G (2920 cm^−1^), and 2G (3210 cm^−1^) [33–35]. The D band corresponds to A_1g_ vibration of carbon rings, which is active only in the presence of defects, thus the magnitude of this band carries information about the disordering of the structure of carbon. In turn, the G band originates from the in-plane stretching of sp^2^ C = C bonds, and it is proportional to crystalline-graphitic character of carbon [33]. The ratio of areas of D and G bands – A_D_/A_G_, was used to estimate the structural ordering of carbon nanofibers, and the exact contributions of the peaks were extracted using Sadezky 5-band model [36, 37]. The A_D_/A_G_ for eCNF/MnO was equal to 1.52 ± 0.19, which is considerably lower than value obtained for non-treated eCNF (2.37 ± 0.61), indicating partial catalytical graphitization of carbon. Furthermore, in the case of nanocomposite, the D band was significantly redshifted and narrowed (FWHM D: 227 ± 4 cm^−1^ → 116 ± 25 cm^−1^), which further confirms the development of crystalline structure and reduction of defects concentration in carbon induced by MnO [33, 38, 39].

#### Chemical composition and state of the surface

**Fig. 3:**
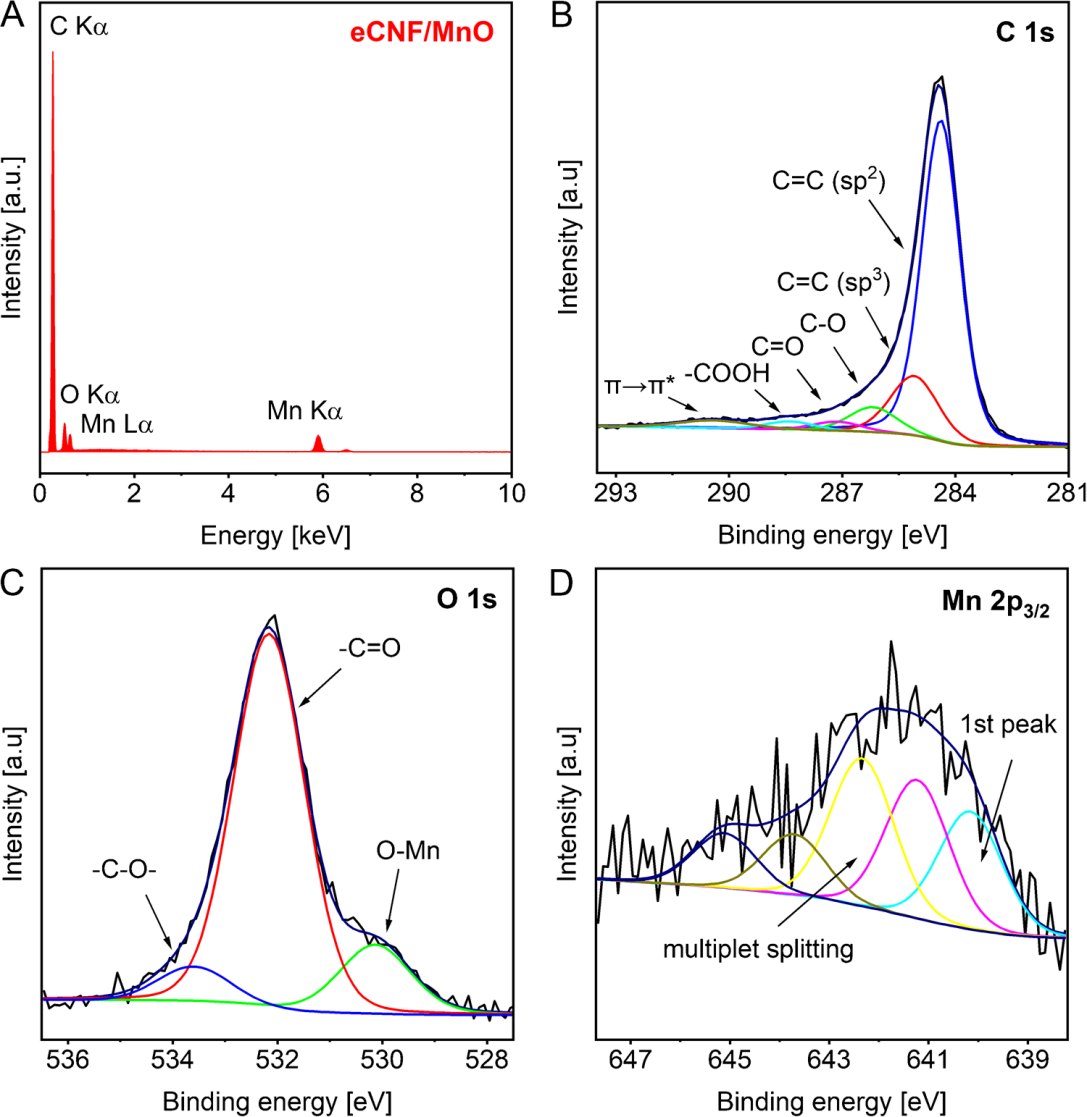
**A** EDS spectra of nanocomposite and XPS core level spectra of **B** C1s, **C** O1s, **D** Mn2p_3/2_ regions

Chemical composition and state of the surface of nanocomposite were analyzed using EDS and XPS, and recorded spectra are shown in Fig. 3. The EDS measurements revealed presence of carbon, oxygen, and manganese, thus testifying inferred chemical composition, and validating the lack of impurities in the obtained nanocomposite. The bulk elemental percentage, as acquired by EDS, equaled to 75.8, 18.2 and 5.9%wt. for C, O, and Mn, respectively. This well agrees with the chemical composition of the surface determined from XPS spectra, which showed the presence of C1s, O1s, Mn 2 s, 2p and 3p photoelectron peaks, and corresponding Auger lines. The elemental composition of the surface calculated from the spectra was 83.4, 13.9 and 2.8%wt. for C, O and Mn, respectively. The core level spectra were subjected to deconvolution procedure, what allowed us to gain deeper insight into the distinct chemical states for each element. The C1s spectrum was fitted with six components assigned to C = C sp^2^ bonds (284.4 eV), C–C sp^3^ bonds (285.0 eV), C-O bonds (286.2 eV), C = O bonds (287.2 eV), COOH groups (288.4), and π → π* shake-up (290.4 eV) [40–42]. Calculated share of sp^2^ carbon in the C1s peak was 72.7%, which demonstrate an unusually strong graphitic character of obtained eCNF [43, 44]. The O1s line was fitted with three peaks corresponding to Mn–O bonds (530.2 eV), C = O (532.2 eV), and C-O bonds (533.6 eV) [42]. The Mn 2p_3/2_ region was fitted with five components, with the first line centered at 640.2 eV, characteristic for Mn^2+^ in MnO [42]. The four peaks with energies 641.2, 642.2, 643.6, 645.1 eV are originating from the multiplet splitting phenomena, and well agree with the positions observed in case of MnO [45, 46].

### Electrochemical characterization of eCNF/MnO/GC electrode

The electrochemical behavior of nanocomposite modification layer on GC electrode was investigated. In this case, the EIS measurements were performed in order to designate the capacitance (C_eff_) and charge transfer resistance (R_ct_) on both electrodes. To calculate the capacitance parameter, the data obtained from ZSimpWin software were used, according to the following equation:

1 $${C}_{eff}={Y0}^{{~}^{1}\!\left/ \!{~}_{N}\right.}\bullet {\left[\frac{1}{{R}_{s}}+\frac{1}{{R}_{p}}\right]}^{\frac{N-1}{N}}$$

C_eff_ and R_ct_ values are presented in the Table 1.

**Table 1:** The capacitance and charge transfer resistance parameters values obtained for both the modified and unmodified electrode

	GC	eCNF/MnO/GC
C, µF	0.037	0.268
R_ct_, kΩ	0.98	0.82

However, the capacitance parameter is higher in case of bare glassy carbon electrode, the charge transfer resistance value is lower for the modified electrode. Considering the results obtained from EIS measurement, it is possible to say that the proposed modification layer consisted of the eCNF/MnO let to obtain more favorable properties of the working electrode for the processes of electrooxidation of the depolarizers in comparison with the glassy carbon electrode, which may result in the better analytical performance.

### Voltammetric characterization of thiethylperazine on eCNF/MnO/GC electrode

**Fig. 4:**
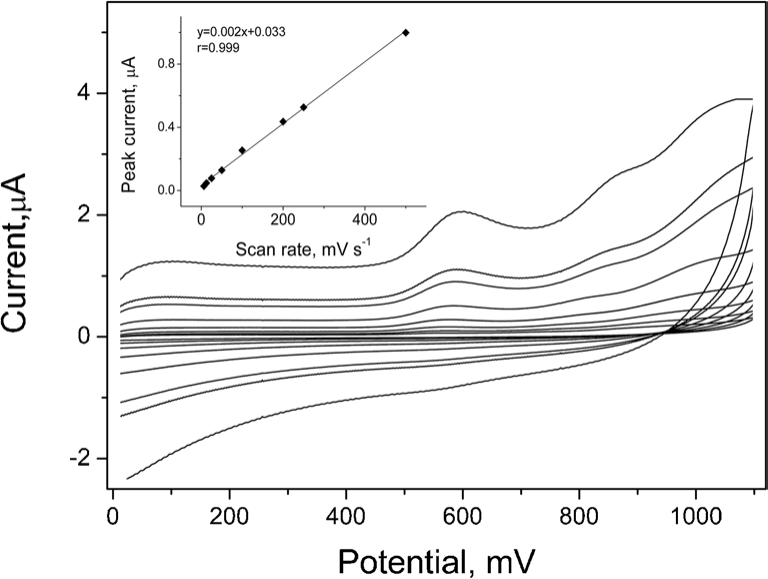
Cyclic voltammograms of 5 μmol L^−1^ thiethylperazine measured in the 0.05 mol L^−1^ acetate buffer pH 5.6 on the eCNF/MnO/GC electrode. The scan rate values were as follows: 6.3, 12.5, 25, 50, 100, 200, 250, and 500 mV s.^−1^

The behavior of thiethylperazine on the surface of eCNF/MnO/GC electrode was investigated using Linear Sweep Voltammetry technique. Measurements were performed in the 0.05 mol L^−1^ acetate buffer with pH 5.6, for the scan rate values from the range 6.3 to 500 mV s^−1^. To clarify the mechanism of reaction taking place on the surface of modified electrode, dependences of the peak current on the scan rate and on the square root of the scan rate value were plotted. The linear dependence was obtained for the scan rate values; therefore, it is possible to say that the considered reaction is controlled by adsorption. The obtained voltammograms are presented in Fig. 4.

In order to gather more information of the THP electrode process, including the number of electrons exchanged, the dependence of the nα coefficient was calculated using the following equation [47]:

2 $$n\alpha =\frac{0.048}{\left|{E}_{p}-{E}_{{p}^{{~}^{1}\!\left/ \!{~}_{2}\right.}}\right|}$$

**Scheme 1:**
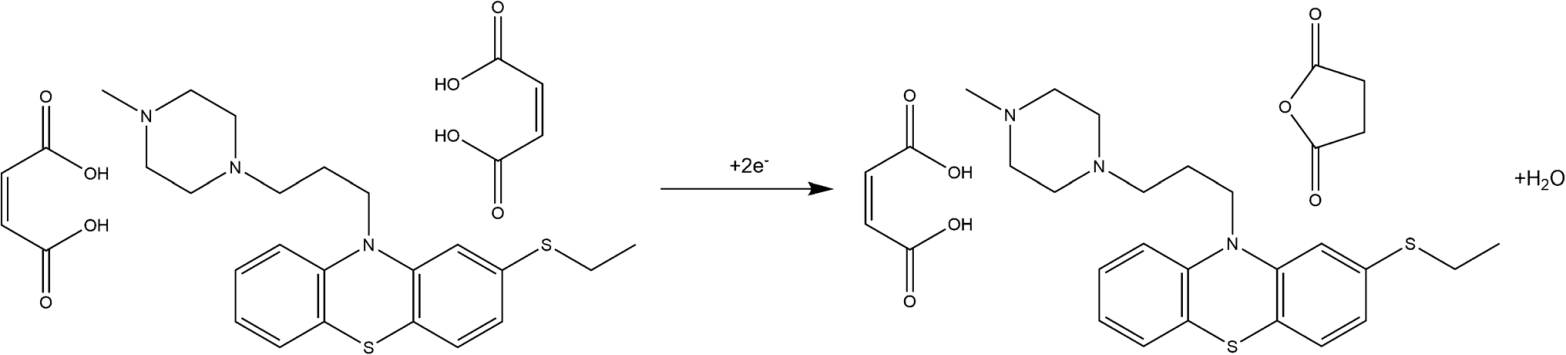
Proposed reaction of thiethylperazine maleate oxidation on the eCNF/MnO/GC electrode

The calculated nα value was equal to 0.87, which considering α as typical 0.5, gives the result of 2 electrons exchanged in the THP oxidation reaction on the eCNF/MnO/GC electrode. Proposed reaction mechanism of THP oxidation is presented in Scheme 1.

Also, the dependence of the pH of acetate buffer on THP was investigated. In this respect, buffers with concentration 0.05 mol L^−1^ and pH values of 3.8, 4.5, 5.0, 5.6, and 6.0 were used as a supporting electrolyte and THP peak was registered in each of them (figure not included). The potential of THP peak did not change with the change of the pH value; therefore, it is possible to say, that protons are not taking part in the process of its electrochemical oxidation on the surface of eCNF/MnO/GC electrode. The value of THP peak current was the best in the buffer with pH value of 5.6; therefore, this buffer was chosen for high sensitive THP determination.

### Influence of the DPV parameters on thiethylperazine peak

One of the most important part of conducting the voltammetric measurements is to choose optimal instrumental parameters characteristic for used technique. In this study, differential pulse voltammetry is chosen for high sensitive thiethylperazine measurements; therefore, parameters such as pulse potential (dE), step potential (E_s_), waiting, and sampling time (t_w_ and t_s_ respectively) were investigated in the wide range of values. The final values chosen for measurements were as follows: dE = 50 mV, E_s_ = 5 mV, t_p_ = t_w_ = 10 ms.

### Influence of the supporting electrolyte on the thiethylperazine peak

In order to provide most suitable conditions for high sensitive THP measurements, different compositions of supporting electrolyte were tested in this respect. The THP behavior was checked in solutions such as 0.1 mol L^−1^ phosphate buffers (pH 5.5 and 7.0), 0.05 mol L^−1^ acetate buffers (pH 3.8, 4.5, 5.6 and 6.0), 0.1 mol L^−1^ sodium tetraborate solution (pH 9.3), 0.05 mol L^−1^ hydrochloric acid (pH 1.3), and 0.035 mol L^−1^ acetate acid (pH 3.2). Signals obtained in the 0.05 mol L^−1^ acetate buffer pH 5.6 exhibited the best properties considering the signal—background relation and stability of the measurements; therefore, this supporting electrolyte was chosen for further studies.

### Influence of preconcentration time and potential on thiethylperazine peak

The influence of the preconcentration time and potential on the thiethylperazine peak current value was tested in order to achieve high sensitivity and low detection limit of the analyte. The preconcentration potential was tested in the wide range, starting from -500 to 500 mV (fig. not included). The preconcentration potential within the range 200 to 500 mV strongly influenced the THP peak, which is present at the potential of 605 mV. The E_acc_ values from the range -500 to 100 mV only slightly affect the THP peak; therefore, the preconcentration potential of 0 mV was chosen for its high sensitive measurements to provide stability and repeatability of the registered data.

**Fig. 5:**
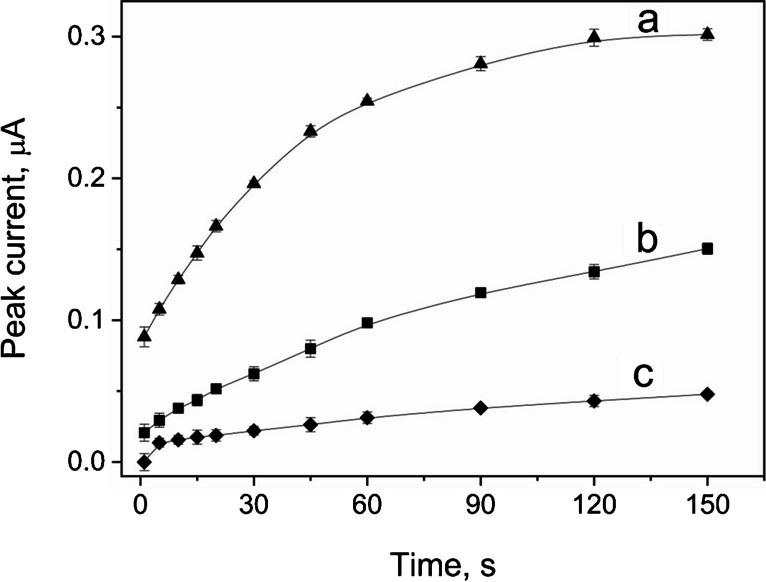
Dependence of the thiethylperazine peak current on the value of preconcentration time. THP concentrations are as follows: **a** 0.5 μmol L^−1^; **b** 0.2 μmol L^−1^; **c** 0.05 μmol L.^−1^. Other instrumental parameters as in point 8

The dependences between thiethylperazine peak and preconcentration time are presented in Fig. 5 for three different THP concentrations. As can be seen, THP peak show the tendencies to increase in the value with the elongation of the preconcentration time on the surface of the eCNF/MnO/GC electrode. After reaching the maximum value, the THP current remains stable despite still increasing time.

### Interferences

Influence of different organic and non-organic interferents on thiethylperazine peak was investigated as the next step of measurements. The results showed that the additions of the following nonorganic substances, such as Zn (II), Al (III), Pb (II) (20 μmol L^−1^), Mg (II), Ca (II), K, SO_4_^2+^, NO_3_^−^, NH_4_^+^, Cl^−^ (1 mmol L^−1^), and CO_3_^2−^ (0.2 mmol L^−1^), did not cause a difference in the THP peak current and potential value. However, the difference in the THP current was observed after addition of 20 μmol L^−1^ Fe (III) (decrease of 22%), and the difference in the THP peak potential was observed for addition of 20 μmol L^−1^ Cu (II), where the peak shifted towards less positive potentials (10 mV of change). Moreover, the addition of organic compounds that may possibly occur in pharmaceutical samples or body fluids was tested. The addition of glucose (0.1 mmol L^−1^), lactose monohydrate (60 μmol L^−1^), uric acid (5 μmol L^−1^), aspartame, caffeine, microcrystalline cellulose, and magnesium stearate (20 μmol L^−1^) did not interfere with the THP peak.

### Analytical performance

**Fig. 6:**
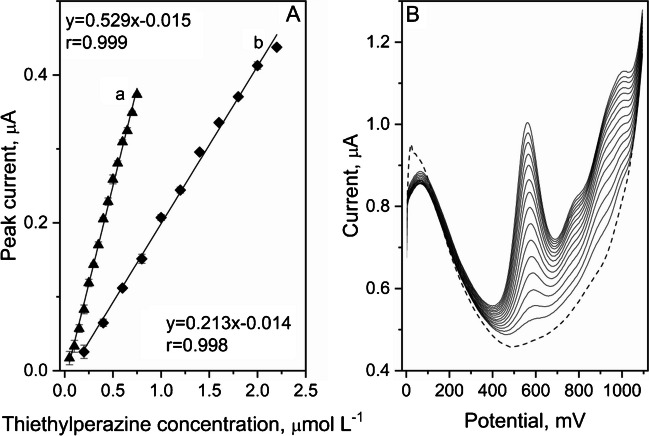
DPV thiethylperazine calibration plots registered for the preconcentration times (a) 30 s and (b) 5 s in 0.05 mol L^−1^ acetate buffer (pH 5.6) (**A**) and corresponding voltammograms obtained for preconcentration time 30 s in the concentration range from 0.05 to 0.75 μmol L^−1^ (**B**). Blank marked as dashed line. Other instrumental parameters as in point 2.7

According to the current literature, the voltammetric determination of thiethylperazine was not performed before. Thiethylperazine voltammograms were registered in the concentration range from 0.05 to 2.2 μmol L^−1^ using DPV technique. Obtained calibration plots and corresponding voltammograms are presented in Fig. 6. The THP linear response covered the whole tested concentration range, with two intervals: 0.05 – 0.75 μmol L^−1^ (slope: 0.21 ± 0.0041 [μA (10^–6^ mol L^−1^)^−1^] for the 30 s of preconcentration, intercept: -0.014 ± 0.0055 μA, r = 0.998) and 0.2 – 2.2 μmol L^−1^ (slope: 0.05 ± 0.0008 [μA (10^–6^ mol L^−1^)^−1^], intercept: -0.015 ± 0.0036 μA, r = 0.998) for the 5 s of preconcentration. On the basis of the obtained results, limit of detection was calculated as 6.3 nmol L^−1^, using the equation LOD = 3.3 s/b, where s was the value of standard deviation from the blank and b was the slope of calibration line. In Table 2, the comparison of the THP LOD obtained by DPV technique with other analytical methods is presented. Exceeding the upper limit of the linearity range may result in a loss of linearity of the measurements due to the blocking of the electrode's active surfaces by the depolarizer.

**Table 2:** Comparison of thiethylperazine determination methods

Method	Linear range	LOD	Reference
Fluorimetry	1 – 10 µg mL^−1^	NI	[7]
Spectrophotometry	5 – 30 µg mL^−1^	NI	[8]
Spectrophotometry	3 – 40 µg mL^−1^	NI	[49]
MALDI-TOF MS	NI	100 pg mL^−1^	[9]
HPLC/ES tandem MS	2 – 40 ng mL^−1^	0.5 ng mL^−1^	[10]
HPLC	9 – 27 µg mL^−1^	0.661 µg mL^−1^	[11]
DPV	0.05 – 2.2 µmol L^−1^	6.3 nmol L^−1^	This work

*NI*, no information; *MALDI-TOF MS*, matrix-assisted laser desorption ionization time-of-flight mass spectrometry; *HPLC/ES tandem MS*, high-performance liquid chromatography electrospray tandem mass spectrometry; *HPLC*, high-performance liquid chromatography; *DPV*, differential pulse voltammetry

The repeatability of the modified electrode was calculated and expressed as RSD, using the equation RSD = 100*s/x, where s is the sample standard deviation and x is sample signal mean value. The RSD value was specified for three different concentrations of THP: 0.1, 1.0, and 2.0 μmol L^−1^ for 7 consecutive voltammograms registrations. The RSD obtained on the modified electrode for the THP concentration of 0.1 μmol L^−1^ was excellent, with the value 1.3%. For higher THP concentration such as 1.0 and 2.0 μmol L^−1^ the RSD values was very good, equal to 1.0 and 1.2% respectively. Also, the long-term stability of the sensor was determined. During the performed measurements, the modified electrode was used for the time of four weeks without significant changes in the THP signal value; in this time, the number of registered scans was of about 700 voltammograms registrations. Between the measurements days, the electrode was storage in the laboratory and covered by the glass vessel in order to reduce the access of dust to its surface. The precision of the THP determination on the different sensors was also measured and expressed as RSD with the value of 12%.

eCNF/MnO/GC performance as a new voltammetric sensor was used to analyze thiethylperazine concentration in tablet and plasma samples. The samples were prepared according to the procedure from point 2.3 and the THP quantitative analysis was performed using standard addition method. The results of measurements with recovery parameter collected in Table 3 shown great usefulness for THP sensitive analysis in such samples with complex matrix. The obtained data from tablet analysis were in a good agreement with the producer declaration and the calculated recovery parameter in the range from 98 to 103% was good.

**Table 3:** Results of thiethylperazine determination in different samples

Sample	Added, mg	Found ± (SD), mg	Recovery, %
Tablet	0	6.41 ± 0.03	98
	13.0	19.3 ± 0.05	100
	26.0	32.7 ± 0.03	102
	39.0	44.9 ± 0.06	99
Producer declares 6.5 mg thiethylperazine per tablet			
Sample	Added, μmol L^−1^	Found ± (SD), μmol L^−1^	Recovery, %
Plasma (20 × diluted)			
	0	ND	-
	0.20	0.20 ± 0.02	100
	0.40	0.39 ± 0.03	98
	0.60	0.62 ± 0.02	103

*ND*, not detected; *SD*, standard deviation for 3 independent measurements

Also, studies of thiethylperazine biodegradability were performed. Eight tablets containing 6.5 mg each were crushed and placed in the water sample from Rudawa river, collected in the center of Krakow. Water was previously filtered on the hard quantitative filter in order to get rid of solid particles. The suspension was placed in the daylight with the access to the air for the time of four weeks. The THP content was examined using proposed voltammetric assay in the day the experiment started (day 0), and then after 7, 14, 21, and 28 days. The results of the analysis showed that the THP undergoes full degradation in such conditions, after the day 21.

### Flow injection analysis with amperometric and voltammetric detection

**Fig. 7:**
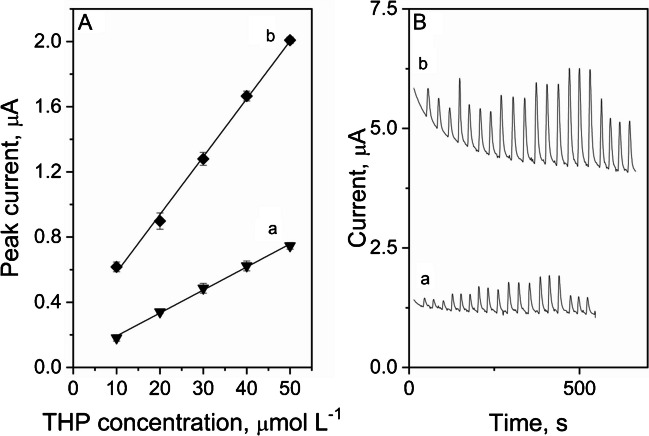
Thiethylperazine calibration plots for unmodified (a) and modified (b) SPC electrode in the concentration range from 1 to 5 μmol L^−1^ with the amperometric parameters of measurements (**A**) and corresponding fiagrams (**B**). All experiments performed under flow injection conditions

The amperometric measurements of thiethylperazine under the flow injection conditions were also performed. The parameters characteristics for the FIA analysis, such as working potential and flow rate, were investigated in order to choose optimal conditions for THP calibration in the amperometric system. Chosen working potential value was 1000 mV, and optimal flow rate was equal to 1.5 mL min^−1^ (figure not included). The calibration of THP was performed both on unmodified and modified with eCNF/MnO SPC electrode, in the concentration range from 1 to 5 μmol L^−1^, and the results of measurements are visualized in Fig. 7. The linear response of analyte was observed in the whole range of tested concentrations, with the slope regression line parameter value of 0.014 μA (10^–6^ mol L^−1^)^−1^ for unmodified and 0.035 μA (10^–6^ mol L^−1^)^−1^ for modified electrode. The RSD of performed amperometric measurements was equal to 2.7% for modified and 4.4% for unmodified electrode, which is a very good result. The accuracy of the method was also calculated, with the results equal to 103% and 95% for unmodified and modified electrode respectively. The content of THP in tablet sample as also measured using FIA method, basing on the calibration graph. Obtained results were in a good agreement with producers declaration; therefore, the usefulness of the method in a quality control analysis was confirmed. According to available literature, eCNF/MnO modified SPCE was used for the first time for thiethylperazine determination in flow analysis condition. The usage of proposed procedure allows to significantly reduce the time of single analysis while maintaining high sensitivity of measurements, which led to the conclusion that proposed assay may be useful for quality control of the THP content in samples. The time of single sample analysis for both amperometric and voltammetric detection was of about 30 s, and the whole calibrations procedures presented in Fig. 7 took about 10 min.

## Conclusions

To date, no sensor has been reported capable of measuring thiethylperazine with an electrochemical methodology with low detection limits, excellent accuracy, and simplicity. Therefore, a new DPV method is proposed using a glassy carbon electrode modified with semi-graphitized carbon nanofibers/MnO nanocomposite. The proposed method allows measurement of THP in concentrations as low as 6.3 nmol L^−1^ (LOD), and the repeatability of the sensor is excellent. The use of the eCNF/MnO/GC advantages in the wide range of linearity, low detection limits, great repeatability of the measurements and stability, which allows one to use such modified electrode for a four weeks without significant sensitivity loss. Another advantage of the presented assay is the ease of electrode preparation and low cost of such sensor. Moreover, on the obtained results, it is possible to say that proposed method is suitable for the determination of THP in complex matrix, such as tablets or human plasma, with great recovery values. Furthermore, it has been proven that thiethylperazine can be measured successfully on SPC electrodes under the flow injection conditions, which implies the possibility of the process automatization. It also allows to decrease the costs of analysis and significantly reduce the time needed for the single analysis.

### Supplementary Information

Below is the link to the electronic supplementary material.

Supplementary file1 (DOCX 76 KB)
