# Supplementary material for: Electrochemical determination of thiethylperazine using semi-graphitized carbon nanofibers-MnO nanocomposite
Source: Mikrochim Acta. 2023 Oct 24;190(11):449. doi: 10.1007/s00604-023-06025-1 (PMC10598083; doi:10.1007/s00604-023-06025-1)
Supplement: Supplementary file 1 — Supplementary file1 (DOCX 76 KB) [file 604_2023_6025_MOESM1_ESM.docx]

**Electronic Supplementary Material**

**Electrochemical determination of thiethylperazine using semi-graphitized carbon nanofibers-MnO nanocomposite**

Joanna Smajdor^1*^, Marcel Zambrzycki^2^, Mateusz Marzec^3^, Beata Paczosa-Bator^1^, Robert Piech^1*^

*^1^ Department of Analytical Chemistry and Biochemistry, Faculty of Materials Science and Ceramics, AGH University of Science and Technology, Al. Mickiewicza, 30-059 Krakow, Poland*

*^2^ Department of Biomaterials and Composites, Faculty of Materials Science and Ceramics, AGH University of Science and Technology, Al. Mickiewicza, 30-059 Krakow, Poland*

*^3^ Surface and Biomaterials Nanoengineering, Academic Centre for Materials and Nanotechnology, AGH University of Science and Technology, Al. Mickiewicza, 30-059 Krakow, Poland*

* Correspondence: [rpiech@agh.edu.pl](mailto:rpiech@agh.edu.pl), [smajdorj@agh.edu.pl](mailto:smajdorj@agh.edu.pl)

**Figure S1.** Raman spectra of manganosite (MnO). RRUFF ID: R060695. Reference: Lafuente B, Downs R T, Yang H, Stone N (2015) The power of databases: the RRUFF project. In: Highlights in Mineralogical Crystallography, T Armbruster and R M Danisi, eds. Berlin, Germany, W. De Gruyter, pp 1-30]
